# Factors associated with health survey response among young employees: a register-based study using online, mailed and telephone interview data collection methods

**DOI:** 10.1186/s12889-020-8241-8

**Published:** 2020-02-05

**Authors:** Tea Lallukka, Olli Pietiläinen, Sauli Jäppinen, Mikko Laaksonen, Jouni Lahti, Ossi Rahkonen

**Affiliations:** 1 Department of Public Health, University of Helsinki, P.O. Box 20, 00014 Helsinki, Finland; 2 Finnish Institute of Occupational Health, Helsinki, Finland; 3 Finnish Centre for Pensions, Helsinki, Finland; 4 The Social Insurance Institution of Finland, Helsinki, Finland

**Keywords:** Mail survey, Online survey, Telephone interview, Young employees, Participation, Response, Socioeconomic factors, Workplace, Health-related factors, Register linkages

## Abstract

**Background:**

Declining response rates are a common challenge to epidemiological research. Response rates further are particularly low among young people. We thus aimed to identify factors associated with health survey response among young employees using different data collection methods.

**Methods:**

We included fully register-based data to identify key socioeconomic, workplace and health-related factors associated with response to a health survey collected via online and mailed questionnaires. Additionally, telephone interviews were conducted for those who had not responded via online or to the mailed survey. The survey data collection was done in autumn 2017 among young employees of the City of Helsinki, Finland (18–39 years, target population *n* = 11,459).

**Results:**

The overall response to the survey was 51.5% (*n* = 5898). The overall findings suggest that differences in the distributions of socioeconomic, workplace and health-related factors between respondents in the online or mailed surveys, or telephone interviews, are relatively minor. Telephone interview respondents were of lower socioeconomic position, which helped improve representativeness of the entire cohort. Despite the general broad representativeness of the data, some socioeconomic and health-related factors contributed to response. Thus, non-respondents were more often men, manual workers, from the lowest income quartile, had part-time jobs, and had more long sickness absence spells. In turn, job contract (permanent or temporary) and employment sector did not affect survey response.

**Conclusions:**

Despite a general representativeness of data of the target population, socioeconomically more disadvantaged and those with long sickness absence, are slightly overrepresented among non-respondents. This suggests that when studying the associations between social factors and health, the associations can be weaker than if complete data were available representing all socioeconomic groups.

## Background

Current challenge in health-related surveys is the declining response rates. Non-response analysis and evidence on factors associated with survey response may help in future data collection, and analyses comparing the target group and the respondents help estimating the representativeness and the generalizability of the findings in similar cohorts. Previous studies have typically reported that male sex, younger age, lower socioeconomic position, and poorer health and health behaviours such as heavier alcohol consumption are key factors associated with non-response [1–4]. Survey respondents’ generally better health is also reflected e.g. as their lower risk of mortality [5].

Several factors may contribute to willingness and motivation to respond in surveys. A recent study using nationally representative data in Finland showed that in the younger age group (29–44 years), the most common reasons not to participate in a health examination were that time or place were found unsuitable (60%) [6]. The study also assessed factors that could enhance survey response and identified financial compensation as the key factor (38%). Also a possibility to choose time and location for examination, physical physician examination/tests, could enhance participation. Only 19% of people reported that they would not participate for any reasons. As response rates have dropped even below 50% particularly in some population subgroups, this suggests that there is still potential to reach more people, with appropriate incentives and flexibility.

Only a few previous studies have had complete register-based data available to accurately assess factors associated with response among those invited to health surveys. Our prior non-response study focusing on a cohort of midlife public sector employees towards the end of their work careers showed that those who did not respond were more likely men, had a lower socioeconomic position and more medically certified sickness absence [4]. However, the cohort did not comprise any younger employees, and the baseline data were collected nearly two decades ago via mailed survey only. Thus, it is not known whether the factors associated with response apply to younger people and early careers, or whether younger employees’ survey response is more strongly determined by other socioeconomic, workplace or health-related factors.

Moreover, it is not well known how survey response patterns are affected by different methods of data collection, i.e. online survey, mailed survey and telephone survey. A recent study from Norway that compared mailed, mailed combined with online option and fully online option for responding found that response rates were above 60% for a group of people receiving only a mailed questionnaire, but only 42% for a separate group of people who only could respond online [7]. The focus of the study was on the measurement of parent experiences with hospital outpatient care for diabetes among children and adolescents with diabetes. In another study from Minnesota, even much lower response rates were achieved using an online only data collection method (14% in the online survey vs. 33% in a mailed survey) [8]. Thus, it appears important to use different data collection methods. As online surveys are more cost-effective and less time-consuming, and require no optical reading after the mailed surveys have been returned, evidence is needed to confirm whether key socioeconomic, workplace or health-related factors are different among respondents to online and mailed surveys. This helps confirm the extent to which the associations between the e.g. social determinants and health outcomes are biased in studies using the data collected with different methods.

We thus aimed to identify socioeconomic, workplace and health-related factors associated with survey response among young employees in Finland using different data collection methods (mailed survey, online survey, and a short telephone interview).

## Methods

### Target population, survey and online data and telephone interviews

#### Description of the target population

This Young Helsinki Health Study collected in autumn 2017, is a new extension of the established Helsinki Health Study, a cohort study following midlife and ageing employees of the City of Helsinki since 2000 [4]. Our target population included 11,459 young employees (18–39 years of age) of the City of Helsinki, Finland, who were born in 1978 or later. Additionally, we only included those who could be reached by mail in Finland and had a job contract of at least 50% of regular work hours per week. The contract further had to have lasted at least four months before the data collection began, since a typical probation period is four months in the City of Helsinki. These criteria were applied to exclude e.g. temporary employees and people working only few hours for the City of Helsinki. They also largely follow data collection of other occupational cohorts in Finland [9]. We next describe how the data were collected using different data collection methods (online and mailed survey and telephone interviews), and describe consent giving to register linkages. Consent does not apply to this fully register-based study, but it is needed in all following studies using these survey data with register linkages. In the last part of this methods section, we describe all register based factors and methods used in the non-response analyses.

#### Description of the online and mailed survey data collection

The target population was first contacted via office email, if it was available. This group comprised the majority of the target population (*N* = 10,044, 87.7%). The email contained a personal link to the online survey. For those without an office email, we mailed the same questionnaire. With the mailed questionnaire, we provided personal login details (to the online version), so that also those receiving a mailed survey could choose, if they preferred to respond online or via mail. For the mailed option, postage was covered. The respondents were informed that they could respond to the survey during their work time. As a vast majority of people in Finland have smart phones, tablets or laptops with email access, we wanted to promote the opportunities to respond with the most applicable methods for each member of the target population.

For those not responding, we sent online reminders (five to all and one more for those who had started to respond but had not completed or sent their questionnaires) and mailed reminders (two), in one week or two weeks intervals. A mailed reminder also included a full questionnaire. This was mailed to all who had not yet responded since some of those with an office email are never or seldom using it. The reminder questionnaire again included personal login details to respond online. Thus, throughout the data collection, it was possible to choose to respond using either the mailed survey or online.

The online questionnaire could be responded in Finnish, Swedish, English or Russian, and the language could be chosen after opening the online survey. We used translation services and translated versions of the measures and questions, to make sure that the questions on different languages are the same. The official work language in the City of Helsinki is Finnish, but as there are also migrants working within the City of Helsinki, we wanted to provide the opportunity to respond with a different language, to promote response in all groups. It was also possible to switch language during responding to the questions. The portal did not save the language used for answering. Thus, it is not possible to report exactly how many used a language other than Finnish. Based on the open-ended questions, answering using another language than Finnish was rare.

The mailed surveys were in Finnish but the respondents were informed about the option to answer using different languages online, or they could ask a survey to be mailed to them in their chosen language. No one asked the survey to be mailed in another language.

#### Telephone interviews

For those who had not answered in two months and had a phone number available, we made a telephone interview. It included 20 most relevant questions of the full survey about health behaviours and working conditions, i.e., key factors associated with work disability and health that are not available in the national registers, and a question about consent to link the survey to national registers.

Telephone number was available for 3266 of the remaining non-respondents, but as it remained possible to respond via mail or online after the interviews were started, we received 311 full questionnaires during the telephone interview process. Thus, phone interviews concerned 2955 members of the target population. Altogether 787 interviews were completed. The most common reason for non-response was that the call was not answered (*n* = 1032). Of those who answered, 779 refused the interview. Other reasons for not succeeding to interview were rarer, for example a wrong number (*n* = 46), or number not in use (*n* = 19).

The calls were made by trained interviewers, to make sure that each survey was done following same guidelines and principles, not leading the respondents in any way.

### Informed consent for register linkages

All respondents were further asked to give their informed consent based on information provided in the cover letter and other required documents, and based on the consent, their survey responses can be retrospectively and prospectively linked to administrative national registers including Statistics Finland, Finnish Centre for Pensions, the Social Insurance Institution of Finland, National Institute for Health and Welfare and the City of Helsinki Personnel register. Separate permission to get the data are applied from each of the register data holders. Consent to link survey data to the register data was provided by 83% of women and 80% of men. However, in this current study about the factors associated with response, only City of Helsinki personnel register data were used, without any linkage to the survey responses.

**Fig. 1:**
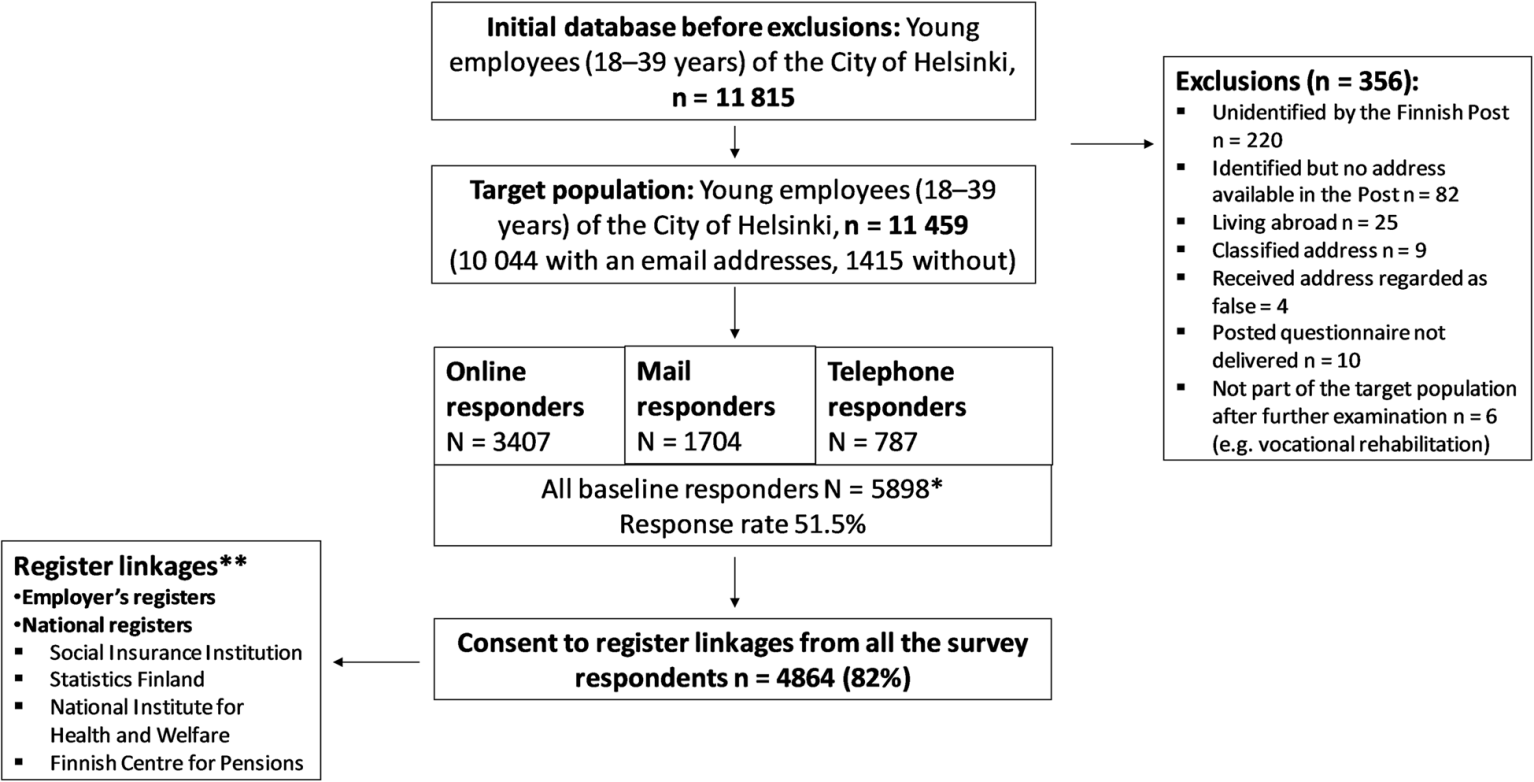
Data collection and respondents of the Young Helsinki Health Study cohort in the different data collection methods. *A vast majority of responses were collected via online surveys. Surveys were mailed to those without an email address and as reminders to all. All mail survey recipients could also reply online, using their personal login details provided with the mailed survey. Finally, telephone interviews were conducted among those who did not respond after reminders, and had an unclassified telephone number (*n* = 3266). **Based on permissions from the register data holders

Data collection is illustrated in more detail in Fig. 1.

### Factors associated with survey response

In analyzing the factors associated with survey response, all variables were derived from the registers of the City of Helsinki. We did not use any survey data in the analyses.

#### Sociodemographic and socioeconomic factors

We used sex, age, occupational class and income as socioeconomic factors associated with survey response. Age was classified into four categories; 18–25 years (reference), 25–29 years, 30–34 years and 35–39 years. There were very few (*n* = 24) employees below 20 years, and that is a reason why they were merged with the 20 to 24 year-olds.

Occupational class was also divided into four groups, following previous procedures within the City of Helsinki [4, 10]: managers and professionals (reference), semi-professionals, routine-non manuals, and manual workers. Income (salary) was divided into quartiles, using the highest quartile as the reference category.

#### Workplace-related factors

Workplace-related factors comprised employment sector, contract type, having a full-time vs. part-time employment, work arrangements and years employed by the City of Helsinki, Finland.

Employment sector referred to social and health care (reference), education or other. Contract type was dichotomized into permanent (reference) and temporary. We distinguished between full-time (reference) and part-time jobs. Shift work referred to having a day time job (reference) or shift work or an undetermined job type. Finally, years employed by the City of Helsinki at the time of the data collection were computed as a difference between the date when the job contract began and the date when the data were drawn and classified into four groups: less than 1 year, 1–5 years, more than 5 years, or unknown (ca 1%).

#### Health-related factors

As health-related factors we used information about sickness absence both before and after the data collection started, to reflect health status of the respondents. Sickness absence was measured for two periods. First, for the six months period before 18 Sep 2017, i.e., the date when the surveys were first mailed or emailed, and second during the main data collection period between 18 September 2017 through 31 October 2017. Sickness absence during data collection was assumed to affect participation in a survey related to work that was sent to a work email and allowed to be filled in during the office hours. We further distinguished between the severity of the sickness absence based on the length of absence. Those with no sickness absence served as a reference category, while other groups comprised self-certified sickness absence of 1–3 days, and medically certified sickness absence of 4–14 days and 15 days or more. Same classifications were used for both sickness absence before and during the data collection.

### Ethical approval

The study was ethically approved by the City of Helsinki and Faculty of Medicine, University of Helsinki ethical committees.

### Statistical analyses

We first examined distributions of socioeconomic, workplace and health-related factors among the respondents, as compared to those of the target population. As a statistical test for differences in the distributions (expected and observed) we used the chi-squared (χ2) test (*p*-values for distributions). Second, we compared the distributions of socioeconomic, workplace and health-related factors among respondents to the online, mailed and telephone surveys. Third, we modelled the associations between socioeconomic-, workplace and health related factors and survey response using log-binomial regression models (rate ratios, RR and their 95% confidence intervals, 95% CI). The model was chosen to display the concrete differences in response rates between groups. Model 1 was for bivariate associations (between each socioeconomic, workplace and health-related factor and survey response as the outcome), while Model 2 was adjusted for all socioeconomic and workplace factors simultaneously. Model 3 was a full model, including all variables from Model 2, as well as sickness absence from 6 months before and during the data collection. Thus, the models first show the separate effects of each factor and then mutually adjust for all factors, to confirm, which factors remain associated with the outcome (survey response), after the other factors have been adjusted for. All the analyses were done using the R software.

## Results

Altogether 3407 women and men responded online, 1704 via a mailed survey and 787 in a telephone interview (shorter version of the survey). Altogether, response rate was 51.5% (5898 respondents of the 11,459 belonging to the target population).

Of all the respondents, 79% were women and 21% men, which reflects the sex distribution of the target population (Table 1), although the proportion of men is slightly higher in the target population (23%, *p*-value 0.011). Differences in the distributions of socioeconomic, workplace and health-related factors between the target population and the respondents were in general very small. Thus, the overall key result is that the data broadly represent the target population. However, there were some differences between respondents and non-respondents, and they are summed here and in more detail in the tables.

**Table 1:** Distributions of socioeconomic, workplace and health-related factors among respondents to online and mailed surveys and telephone interviews as compared to the target population (under 40-year-old employees of the City of Helsinki, Finland)

	Target population		Respondents		Statistical test for difference (chi-squared)	Response rate (95% CI)
	N	%	N	%		
Sex						
Women	8801	77	4631	79	0.011	53 (52, 54)
Men	2658	23	1267	21		48 (46, 50)
Age						
18–24 years	974	8	406	7	0.002	42 (39, 45)
25–29 years	3036	26	1559	26		51 (50, 53)
30–34 years	3835	33	2003	34		52 (51, 54)
35–39 years	3614	32	1930	33		53 (52, 55)
Occupational class						
Managers and professionals	2778	24	1606	27	< 0.0001	58 (56, 60)
Semi-professionals	3212	28	1871	32		58 (57, 60)
Routine non-manuals	4307	38	1982	34		46 (45, 48)
Manual workers	1162	10	439	7		38 (35, 41)
Income						
Highest quartile	2866	25	1690	29	< 0.0001	59 (57, 61)
3rd	2937	26	1668	28		57 (55, 59)
2nd	2792	24	1297	22		46 (45, 48)
Lowest quartile	2864	25	1243	21		43 (42, 45)
Employment sector						
Social and health care	4906	43	2546	43	0.886	52 (50, 53)
Education	3964	35	2035	35		51 (50, 53)
Other	2589	23	1317	22		51 (49, 53)
Contract type						
Permanent	3074	27	1566	27	0.712	51 (49, 53)
Temporary	8385	73	4332	73		52 (51, 53)
Job type						
Full time	10,158	89	5279	90	0.093	52 (51, 53)
Part-time	1301	11	619	10		48 (45, 50)
Work arrangement						
Day time	7996	70	4245	72	0.011	53 (52, 54)
Shift work	3414	30	1629	28		48 (46, 49)
Undetermined	49	0	24	0		49 (34, 64)
Years employed by the City of Helsinki^*^						
less than 1 year	1542	13	756	13	0.327	49 (47, 52)
1–5 years	5220	46	2737	46		52 (51, 54)
more than 5 years	4627	40	2378	40		51 (50, 53)
unknown	70	1	27	0		39 (27, 51)
Sickness absence (6 months before the data collection)						
No sick leave spells	5059	44	2577	44	0.008	51 (50, 52)
From 1 to 3 days	2742	24	1521	26		55 (54, 57)
From 4 to 14 days	2682	23	1364	23		51 (49, 53)
15 days or more	976	9	436	7		45 (42, 48)
Sickness absence (during the data collection = 18 Sep–31 Oct 2017)						
No sick leave spells	7893	69	4046	69	0.014	51 (50, 52)
From 1 to 3 days	2216	19	1190	20		54 (52, 56)
From 4 to 14 days	1081	9	565	10		52 (49, 55)
15 days or more	269	2	97	2		36 (30, 42)
Total numbers	11,459	100	5898	100		51 (51, 52)

^*^computed from the date when the job contract began and the date when data were made

Response rates somewhat varied by the examined socioeconomic, workplace and health-related factors. Women had a higher response rate (53, 95% CI 52–54%) than men (48, 95% CI 46–50%). Additionally, those younger than 25 years were less likely to respond as compared to employees from 25 to 39 years. Other age groups’ response rates did not differ.

The highest response rates (59, 95% CI 57–61%) were found among people belonging to the highest income quartile, and among semi-professionals (58, 95% CI 57–60%) and managers and professionals (58, 95% CI 56–60%). The response rates were lowest among people with long sickness absence (15 days or more) during the data collection (36, 95% CI 30–42%), and among manual workers (38, 95% CI 35–41%). Otherwise, differences were small.

In our additional analyses, we stratified the data by sex, to confirm if the factors associated with survey response are same for women and men (data not shown). The results were similar, and that is why only pooled response rates are shown.

For example, occupational class affected the survey response among both women and men, with manual workers being less likely to respond as compared to the managers (39% vs. 59% among women, and 37% vs. 55% among men). Also income played a similar role for both women and men, with those belonging the highest income quartile having the highest response rates (60% among women and 56% among men). Finally, both women and men who had long sickness absence (lasting 15 days or more) during the period of six months before the survey were less likely to respond. However, shorter spells did not affect survey response. Similarly, those with long sickness absence during the data collection period had the lowest response rates among both women and men.

Table 2 displays the distributions of socioeconomic, workplace and health-related factors separately for online survey and mailed survey respondents, as well as telephone interview respondents. We also merged online and mailed survey respondents, since the surveys were the same, while telephone interview only comprised part of the questions. A clear sex-difference was observed, as men were underrepresented among the mailed survey respondents (16%), but overrepresented in the telephone interview (30%). When the surveys are combined, sex distribution is closer to that of the target population. In all, distributions of the socioeconomic and workplace factors were very similar among the online and mail survey respondents, while the respondents of the telephone survey were more likely of lower socioeconomic position, such as lower occupational class, or lower income, and also more likely to have a part-time job. Thus, adding the telephone survey in the data made the data more representative, and the distributions of the socioeconomic factors closer to the target population.

**Table 2:** Distributions of socioeconomic, workplace and health-related factors associated with survey response among under 40-year-old employees of the City of Helsinki, Finland in 2017 using online, mailed and telephone survey data collection methods

	Online survey		Postal survey		Online + postal		Telephone interview		Non-respondents	
	N	%	N	%	N	%	N	%	N	%
Sex										
Women	2643	78	1436	84	4079	80	552	70	4170	75
Men	764	22	268	16	1032	20	235	30	1391	25
Age										
18–24 years	221	6	106	6	327	6	79	10	568	10
25–29 years	890	26	486	29	1376	27	183	23	1477	27
30–34 years	1137	33	605	36	1742	34	261	33	1832	33
35–39 years	1159	34	507	30	1666	33	264	34	1684	30
Occupational class										
Managers and professionals	945	28	470	28	1415	28	191	24	1172	21
Semi-professionals	1153	34	521	31	1674	33	197	25	1341	24
Routine non-manuals	1116	33	568	33	1684	33	298	38	2325	42
Manual workers	193	6	145	9	338	7	101	13	723	13
Income										
Highest quartile	1035	30	456	27	1491	29	199	25	1176	21
3rd	1008	30	477	28	1485	29	183	23	1269	23
2nd	716	21	393	23	1109	22	188	24	1495	27
Lowest quartile	648	19	378	22	1026	20	217	28	1621	29
Employment sector										
Social and health care	1606	47	685	40	2291	45	255	32	2360	42
Education	960	28	744	44	1704	33	331	42	1929	35
Other	841	25	275	16	1116	22	201	26	1272	23
Contract type										
Permanent	938	28	437	26	1375	27	191	24	1508	27
Temporary	2469	72	1267	74	3736	73	596	76	4053	73
Job type										
Full time	3066	90	1530	90	4596	90	683	87	4879	88
Part-time	341	10	174	10	515	10	104	13	682	12
Work arrangement										
Day time	2421	71	1253	74	3674	72	571	73	3751	67
Shift work	978	29	439	26	1417	28	212	27	1785	32
Undetermined	8	0	12	1	20	0	4	1	25	0
Years employed by the City of Helsinki^*^										
less than 1 year	406	12	239	14	645	13	111	14	786	14
1–5 years	1608	47	762	45	2370	46	367	47	2483	45
more than 5 years	1384	41	695	41	2079	41	299	38	2249	40
unknown	9	0	8	0	17	0	10	1	43	1
Sickness absence (6 months before the data collection)										
No sick leave spells	1389	41	807	47	2196	43	381	48	2482	45
From 1 to 3 days	924	27	421	25	1345	26	176	22	1221	22
From 4 to 14 days	849	25	364	21	1213	24	151	19	1318	24
15 days or more	245	7	112	7	357	7	79	10	540	10
Sickness absence (during the data collection = 18 Sep–31 Oct 2017)										
No sick leave spells	2265	66	1222	72	3487	68	559	71	3847	69
From 1 to 3 days	750	22	311	18	1061	21	129	16	1026	18
From 4 to 14 days	355	10	133	8	488	10	77	10	516	9
15 days or more	37	1	38	2	75	1	22	3	172	3
Total numbers	3407	100	1704	100	5111	100	787	100	5561	100

^*^computed from the date when the job contract began and the date when data were made

In general, unadjusted rate ratios (Table 3, model 1) showed that older employees were more likely to respond, while those with a lower occupational class and lower income, those doing shift work, and those with medically certified long sickness absence spells (15 days or more) particularly during the main data collection period were less likely to respond in the survey. Mutual adjustment for all sociodemographic variables (Model 2) affected most of the estimates, and they e.g. attenuated (age, occupational class, sickness absence), or changed direction (part-time job). After full adjustments (model 3), being a manual worker, lower income (salary), working within the employment sector ‘education’, and long sickness absence during the main data collection period were associated with lower response rates whereas the rates were higher for older employees, semi-professionals, and those with part-time work. Again, the factors were similar for women and men (data not shown).

**Table 3:** Socioeconomic, workplace and health-related factors associated with survey response among under 40-year-old employees of the City of Helsinki, Finland in 2017 (rate ratios, RR, and their 95% confidence intervals, 95% CI)

	Model 1 (bivariate associations)	Model 2 (all sociodemographic variables adjusted for)	Model 3 (Model 2 and sickness absence adjusted for)
	RR (95% CI)	RR (95% CI)	RR (95% CI)
Sex			
Women	1.0	1.0	1.0
Men	0.91 (0.87, 0.95)	0.92 (0.88, 0.96)	0.91 (0.87, 0.96)
Age			
18–24 years	1.0	1.0	1.0
25–29 years	1.23 (1.13, 1.34)	1.11 (1.02, 1.21)	1.11 (1.02, 1.21)
30–34 years	1.25 (1.16, 1.36)	1.09 (1.00, 1.19)	1.10 (1.01, 1.20)
35–39 years	1.28 (1.18, 1.39)	1.11 (1.01, 1.21)	1.11 (1.02, 1.21)
Occupational class			
Managers and professionals	1.0	1.0	1.0
Semi-professionals	1.01 (0.97, 1.05)	1.11 (1.04, 1.18)	1.11 (1.04, 1.18)
Routine non-manuals	0.80 (0.76, 0.83)	1.02 (0.94, 1.10)	1.01 (0.94, 1.10)
Manual workers	0.65 (0.60, 0.71)	0.74 (0.67, 0.83)	0.74 (0.67, 0.83)
Income			
Highest quartile	1.0	1.0	1.0
3rd	0.96 (0.92, 1.01)	0.88 (0.83, 0.94)	0.88 (0.83, 0.94)
2nd	0.79 (0.75, 0.83)	0.80 (0.74, 0.86)	0.79 (0.73, 0.86)
Lowest quartile	0.74 (0.70, 0.78)	0.75 (0.69, 0.82)	0.75 (0.69, 0.82)
Employment sector			
Social and health care	1.0	1.0	1.0
Education	0.99 (0.95, 1.03)	0.93 (0.88, 0.97)	0.92 (0.88, 0.97)
Other	0.98 (0.94, 1.03)	1.08 (1.02, 1.14)	1.08 (1.02, 1.14)
Contract type			
Permanent	1.0	1.0	1.0
Temporary	0.99 (0.95, 1.03)	0.98 (0.93, 1.02)	0.97 (0.93, 1.01)
Job type			
Full time	1.0	1.0	1.0
Part-time	0.92 (0.86, 0.97)	1.11 (1.03, 1.19)	1.11 (1.03, 1.19)
Work arrangement			
Day time	1.0	1.0	1.0
Shift work	0.90 (0.86, 0.94)	0.90 (0.85, 0.95)	0.90 (0.86, 0.95)
Undetermined	0.92 (0.69, 1.23)	0.73 (0.54, 0.99)	0.74 (0.55, 1.01)
Years employed by the City of Helsinki^*^			
less than 1 year	1.0	1.0	1.0
1–5 years	1.07 (1.01, 1.13)	1.00 (0.95, 1.07)	1.01 (0.95, 1.07)
more than 5 years	1.05 (0.99, 1.11)	0.95 (0.89, 1.01)	0.96 (0.90, 1.02)
unknown	0.79 (0.58, 1.06)	0.84 (0.62, 1.13)	0.86 (0.64, 1.16)
Sickness absence (6 months before the data collection)			
No sick leave spells	1.0		1.0
From 1 to 3 days	1.09 (1.04, 1.14)		1.08 (1.04, 1.13)
From 4 to 14 days	1.00 (0.95, 1.05)		1.03 (0.98, 1.08)
15 days or more	0.88 (0.81, 0.95)		0.96 (0.89, 1.03)
Sickness absence (during the data collection = 18 Sep–31 Oct 2017)			
No sick leave spells	1.0		1.0
From 1 to 3 days	1.05 (1.00, 1.09)		1.05 (1.00, 1.09)
From 4 to 14 days	1.02 (0.96, 1.08)		1.09 (1.03, 1.16)
15 days or more	0.70 (0.60, 0.83)		0.75 (0.64, 0.88)

^*^computed from the date when the job contract began and the date when data were made

## Discussion

### Main findings

This study aimed to identify factors associated with health survey response, focusing on a variety of socioeconomic, workplace and health-related factors among under 40-year-old employees. Moreover, we used different methods of survey data collection to confirm whether factors associated with survey response differed between those who responded online, in a mailed survey or via a short telephone interview. Our main findings show that despite the low response, the data generally represent the target population with respect to all studied socioeconomic, workplace and health-related factors. Older age, higher occupational class and higher income appear the key factors associated with survey response, while people with long sickness absence were less likely to respond. Work-related factors such as having a permanent job did not predict survey response, however, shift workers and those with a part-time employment tended to have lower response rates. Overall, socioeconomic, workplace and health-related characteristics of respondents were largely similar between online and mailed surveys, while telephone survey respondents’ comprised more of those with a lower socioeconomic position, helping balance the overall sample and its representativeness of the target population.

### Interpretation

The main findings suggest that although the overall response rate was relatively low, the respondents broadly represent the target population. However, some differences between survey respondents and the target population were found which should be taken into account when interpreting the results. Overall, due to a relatively high sample size, many *p*-values were significant but most of the differences were small with limited practical or meaningful interpretation. Moreover, the rate ratios were weak, and the patterns of the associations suggest that the overall picture is that the data satisfactorily represent the target population.

Despite the overall picture, some differences are described to help interpret the associations in studies using these or similar data, when studying social determinants of health. For example, those with the socially most disadvantaged situation (manual workers, low income), under 25-year-old employees and men as well as those with medically certified long sickness absence during the main data collection period, were overrepresented among the non-respondents. This means that we likely lost more those doing heavy physical work, and people with a higher likelihood of future sickness absence and ill-health [11–13], since prior sickness absence likely predicts subsequent work disability [14]. Thus, the associations regarding many health outcomes could be diluted and the results are likely to be conservative.

The response rate further is in line with many major recent surveys that all suffer from largely declining response rates during previous decades, with many studies currently having response rates below 50% [15]. For example, in our previous data collection [16] among older employees of the City of Helsinki in 2000–2002, 67% responded at baseline [10], but in general the response rates have been low already at baseline, and decreasing in several established cohorts [3, 17, 18]. However, even with a lower response rates, results suggest only slight underestimation of illness prevalence [19]. Typically, e.g. mortality, nonetheless, is higher among non-respondents [19] and those who drop-out during follow-ups [20].

When the response rate is low, the question about selection bias is a major issue, and to better interpret results, it is crucial to understand the extent of bias. In the current cohort, as in the earlier older cohort [10], those with the most disadvantaged socioeconomic situation, more sickness absence, men and the youngest employees were overrepresented among the non-respondents. The factors associated with survey response were largely similar in all data collection methods. The telephone interview was done last, which could explain why it slightly improved socioeconomic representativeness of the data. Thus, those with a lower socioeconomic position were overrepresented among the non-respondents at the time of the telephone interviews, so they were more likely to be called for an interview. A positive finding was that they were willing to respond in the telephone interviews, improving the representativeness of the cohort.

In our earlier study, we have further shown that survey non-response is unlikely to distort analyses of occupational class inequalities in sickness absence to any major extent, or even those generally addressing health inequalities [21]. However, these non-response analyses were only done among older employees of the City of Helsinki, and data were collected earlier and via mailed surveys only. Another study comprising results from 27 populations in the WHO MONICA Project studied how non-response affects population trend estimates due to different socioeconomic and health profiles between non-respondents and respondents [22]. The study concluded that declining response rates are compromising the accuracy of the estimates, particularly when the response rates are declining. These previous and the current findings show that losing those with the most disadvantaged situation and poorest health (as indicated e.g. by low income and long sickness absence) is a serious issue and challenge that has to be considered carefully. In other words, the implication is that it is likely that the results will be slightly conservative, as those with poorer health (more sickness absence), and lower socioeconomic position are overrepresented among the non-respondents. Thus, having complete register data comprising a variety of key factors related to addressing e.g. inequalities in health to accurately and more objectively assess the representativeness of the data of the target population, is important. Such estimation is important in any subsequent studies using data comprising roughly half of the target population. A better understanding about the differences between respondents and the target population, helps improve interpretations of the results and assess their implications. Moreover, it is crucial to also confirm the contribution of data collection method to the distributions of key variables in health studies, and the efficacy of the reminders to increase the response rates.

A previous study using only online data collection similarly suffered from a very low response rate, but found that reminders help increase response rates [23]. In that study, the response rate increased from initial 23 to 39% after a round of reminders. One might still wonder, if the data collected online is equally valid and reliable as compared to mailed surveys, e.g. regarding response rates but also are there differences in who are more likely to respond online. However, already in 2010, web-based questionnaires were seen as promising and potential future of epidemiology, and the data valid and reliable [24]. The authors concluded that comparisons between traditional and online surveys should confirm, if web-based surveys can replace mailed surveys. In light of some previous studies suggesting that online only survey could lead to particularly low response rates [7, 8], it appears it was a reasonable decision to send a mailed questionnaire to all, in addition to the option to respond online. However, it is of note that as our online and mailed surveys as well as telephone surveys were collected at the same time, and each respondent could choose any method, response rates for different data collection methods are not independent. Thus, they cannot be individually considered or directly compared.

Furthermore, in this study we show social, workplace, and health-related factors associated with survey response overall using different data collection methods. While there are some differences in the characteristics of respondents as compared to the target population, in general all data broadly represent the target population fairly well. It is also expected that there are some differences, because online surveys are more likely filled in by those who use emails and computers or laptops at work. The respondents could respond during the office hours, so this might have encouraged more those, who have access to internet during office hours. It is probable that for example manual workers and those who do not use computers or email at work, did not receive the first invitation that came via office email. Thus, they received a reminder and instructions to fill in the survey online, but might not have done so during their leisure time.

Also earlier studies have quite commonly used telephone interviews to complete data collection [25, 26]. Although not that many questions can be asked during a telephone interview, such interviews still help increase response rates, and may reduce bias and selection. Particularly when studying e.g. social determinants of health, it is important that the data are representative of social hierarchy. Additionally, even with short interviews, it is feasible to gather data on key covariates that are otherwise unavailable from registers, such as health behaviors, sleep and working conditions. Despite single items, they can emerge as important covariates in studies with a register-based follow-up and outcomes.

#### Methodological considerations

The advantage of this study was the opportunity to include a wide range of objective, register-based socioeconomic, workplace and health-related factors associated with survey response, to more accurately assess the quality of the data collected using different methods. The wide range of socioeconomic, workplace and health-related factors is a notable strength, as previous non-response analyses have not been able to focus on such a variety of different factors. Overall, such data are rarely available, and a better understanding of the representativeness of the data significantly improves the opportunities to interpret the results, and assess the effects of non-response e.g. when studying social determinants of health. As the included socioeconomic and health-related factors are known to be associated with health and work disability [27–29], the results of the non-response analyses suggest that studying these factors, and the associations between social factors and health is unlikely seriously distorted due to non-response. Moreover, a strength of the study was the comparison of different data collection methods. Short self-certified sickness absence spells and their role in the non-response has not been typically studied, and thus addressing their contribution to survey response is novel and a strength of this study. A limitation of the study is the lack of survey-based data on other key factors, such as health behaviors, sleep or pain, or data on motivational factors or actual reasons why the respondents chose to return the survey or not. Some respondents gave qualitative feedback explaining their situation, but such data could not be analyzed in this study. These factors would deepen our understanding about non-response and perhaps help better target future surveys to those least likely to take part in health surveys [6]. A further limitation is that we could not compare response rates between different survey methods, as all could choose to participate via online or return a mailed survey.

## Conclusions

In this cohort of young employees with a relatively low response rate, the respondents fairly well represented the target population. However, those with a more advantageous socioeconomic circumstances and less long-term sickness absence were more likely to respond to the survey. The selection by socioeconomic position and health suggests that when using such health survey data, the associations between social determinants and health may be conservative. Furthermore, our study shows that data collection method is unlikely to have any strong impact on the factors associated with survey response. Using a shorter telephone interview can help improve representativeness of the data and increase response rate. The interview can comprise key factors that are unavailable from the registers, and therefore it is a recommended means to increase survey response, if the response rates in full surveys are low.

## Data Availability

The register data used for the study are not publicly shared due to data protection laws. The data can be applied from the data holders, following data protection laws.

## Abbreviations

95% CI: 95% confidence intervals
RR: Rate ratios
